# Macro-Invertebrate Decline in Surface Water Polluted with Imidacloprid

**DOI:** 10.1371/journal.pone.0062374

**Published:** 2013-05-01

**Authors:** Tessa C. Van Dijk, Marja A. Van Staalduinen, Jeroen P. Van der Sluijs

**Affiliations:** Environmental Sciences, Utrecht University, Utrecht, The Netherlands; French National Institute for Agricultural Research (INRA), France

## Abstract

Imidacloprid is one of the most widely used insecticides in the world. Its concentration in surface water exceeds the water quality norms in many parts of the Netherlands. Several studies have demonstrated harmful effects of this neonicotinoid to a wide range of non-target species. Therefore we expected that surface water pollution with imidacloprid would negatively impact aquatic ecosystems. Availability of extensive monitoring data on the abundance of aquatic macro-invertebrate species, and on imidacloprid concentrations in surface water in the Netherlands enabled us to test this hypothesis. Our regression analysis showed a significant negative relationship (*P*<0.001) between macro-invertebrate abundance and imidacloprid concentration for all species pooled. A significant negative relationship was also found for the orders Amphipoda, Basommatophora, Diptera, Ephemeroptera and Isopoda, and for several species separately. The order Odonata had a negative relationship very close to the significance threshold of 0.05 (*P* = 0.051). However, in accordance with previous research, a positive relationship was found for the order Actinedida. We used the monitoring field data to test whether the existing three water quality norms for imidacloprid in the Netherlands are protective in real conditions. Our data show that macrofauna abundance drops sharply between 13 and 67 ng l^−1^. For aquatic ecosystem protection, two of the norms are not protective at all while the strictest norm of 13 ng l^−1^ (MTR) seems somewhat protective. In addition to the existing experimental evidence on the negative effects of imidacloprid on invertebrate life, our study, based on data from large-scale field monitoring during multiple years, shows that serious concern about the far-reaching consequences of the abundant use of imidacloprid for aquatic ecosystems is justified.

## Introduction

When neonicotinoids were introduced as new, systemic, insecticides in the 1990s, they were supposed to be much more efficient than the older generation of insecticides [1]. As a seed treatment they could be used in much lower quantities and they promised to be less polluting to the environment. Seed dressing makes spraying crops with insecticides unnecessary because the active substances are spread to all plant tissues when the plant grows. However, soon after the introduction of this new type of insecticides, concern rose that neonicotinoid residues in pollen and nectar might be harmful to honey bees [1], [2], and several studies have provided supporting evidence for this [3].

Neonicotinoids are neuro-active insecticides which derive their toxicity to target species from acting mainly agonistically on nicotinic acetylcholine receptors (nAChRs) on the post-synaptic membrane [4]–[6]. This means that normal nerve impulses become impaired [7]. Some authors [8] have also indicated some antagonistic action. The binding sites in mammal nAChRs are different from those in insect nAChRs, and the neonicotinoid imidacloprid shows selective toxicity for insects over vertebrates. This partly attributable to a higher affinity of imidacloprid for insect nAChRs compared with their vertebrate counterparts [5].

In short-term (10-day) tests on the effects of imidacloprid [9] on the aquatic worm *Lumbriculus variegatus* a high mortality was observed at the highest concentrations of imidacloprid in the sediments (1 to 5 mg/kg). At lower concentrations (0.05 to 0.5 mg/kg) effects were observed on growth and behaviour of *L. variegatus*. In another test [10] the aquatic invertebrates *Chironomus tentans* and *Hyallella Azteca* were able to recover from a short-term pulse exposure, but a chronic low-level exposure (>1.14 µg l^−1^ for *C. tentans*) to imidacloprid reduced the species survival and growth. Different effects of imidacloprid exposure in an aquatic microcosm experiment were found for two species of stream insects [11]; while the survival of the stonefly, *Pteronarcys dorsata*, was significantly reduced at 48 and 96 mg l^−1^, no significant mortality was found for the cranefly, *Tipula sp.*, although a change in behaviour was observed. In acute toxicity bioassays [12] of imidacloprid to zooplankton crustaceans, the imidacloprid 48-h LC_50_-s for cladocerans (65–133 mg l^−1^) were two orders of magnitude higher than for ostracods (301–715 µg l^−1^). In an acute toxicity test on an amphibian [13] the 48-h LC_50_-s for imidacloprid were found to be 165 mg l^−1^ for tadpoles of *Rana limnocharis* and 219 mg l^−1^ for tadpoles of *Rana nivalis*. The variation in susceptibility among different animal taxa indicates that certain biochemical traits particular to a group of organisms are responsible for a specific level of sensitivity [14].

Long-term alterations in aquatic invertebrate community structure occurred after single pulse contamination of a stream ecosystem with the neonicotinoid insecticide thiacloprid [15]. In other community studies, the caddisfly *Neureclipsis sp.* reacted very sensitively to a single pulse of imidacloprid, and Diptera and Ephemeroptera larvae were affected after repeated pulses [16]. In field mesocosms, zooplankton, benthic, nekton as well as neuston communities exposed to imidacloprid were significantly less abundant than non-treated controls [17].

At low concentrations of neonicotinoid insecticides sub-lethal effects can occur in invertebrates. Given the many limitations of acute toxicity as an indicator for impacts of agrochemicals on aquatic invertebrate communities, the sublethal effects must be considered for a complete and realistic assessment of the long term impact [18]. In a study [19] on the effect of imidacloprid exposure on the mayfly *Epeorus lingimanus* and the aquatic oligochaete, *Lumbriculus variegatus* a reduction of feeding and egestion was found. This indicates physiological and behavioural responses to this insecticide. In an extensive review Desneux et al. found that sub-lethal effects of neonicotinoids may occur on neurophysiology, larval development, molting, adult longevity, immunology, fecundity, sex ratio, mobility, orientation, feeding behaviour, oviposition behaviour, and learning [18]. All these effects have been reported for a wide range of invertebrates and all have a potential to produce population level and community level impacts on ecosystems. In bees an additional sub-lethal effect of imidacloprid was found namely an increased susceptibility to infections and parasites such as *Nosema ceranae* [20]–[22]. This effect seems not related to the immune system but to impairment of grooming and allogrooming, which leads to reduced hygiene in the individual and in the nest, and so gives the pathogens more chance to infect the insects.

Delayed and chronic toxicity to aquatic arthropods were found after exposure to very low concentrations of neonicotinoids in water [23]. Thiacloprid caused delayed lethal and sub-lethal effects after 4 to 12 days following exposure. In order to be able to predict the effects of toxicants and to determine safe levels of concentrations of neonicotinoids and other toxicants for organisms, exposure time should be taken into account [24]. As traditional approaches consider toxic effects at fixed exposure times, a new approach to risk assessment is needed in which the time-dependency of the toxicity is included, because lowering the concentrations only means an increase in the time to effect, which is only limited by the natural lifespan of the (unexposed) organism [24]–[26].

Large-scale use of neonicotinoid insecticides started around 2004, and has rapidly increased to make neonicotinoids the most widely used class of insecticides world-wide [27], [28]. Imidacloprid now ranks second in the global top 10 of agrochemicals [29]. Only a small fraction of the pesticide doses used reaches its intended target. Sur and Stork [30] found that for systemic application via seed coating only 1.6 to 20% of the imidacloprid in the seed coating actually enters the crop to protect it. The remaining 80 to 98.4% of the applied amount ends up in the environment, and can accumulate in soil [31], especially because of its high persistence. There are various ways for imidacloprid to contaminate ground or surface water: by accidental spilling, leaching, overspray or spray-drift. Furthermore, imidacloprid used on grass, turf or hard surfaces such as lawns, golf courses or concrete may contaminate surface water through runoff and drainage [32], [33].

Leaching of pesticides is one of the main mechanisms responsible for the contamination of groundwater and surface water. Felsot found that imidacloprid applied via drip chemigation leached significantly below the emitter depth [34]. The Groundwater Ubiquity Score (GUS) [35] of imidacloprid as calculated from the sorption coefficient (K_oc_) and the soil halftime (DT_50_) amounts to 3.76, indicating a high leaching potential [36]. However, the leaching process is highly variable across different soil types and pesticide formulations [37]. The presence of cracks or other macropores in the soil, or less structured soil can lead to preferential flows that bypass the most chemically and biologically reactive topsoil. Leaching from sandy soils is very high while imidacloprid is less mobile in, but still leaches substantially from, soil with a high organic matter content [38]. Estimated equilibrium partitioning over soil and water gives a soil to water ratio of 1 to 3 (log *P* = 0.57), indicating that most of the imidacloprid tends to end up in the water [39]. Note that this ration can vary with varying organic matter content of the soil [38].

Imidacloprid is generally persistent in water, and not easily biodegradable [31]. It is likely to remain in the water column in aquatic systems, and has an aerobic sediment and water half-life time of 30 to 162 days [36], [40]. At pH values corresponding to environmental conditions, imidacloprid is stable to hydrolysis, but it can be rapidly degraded photolytically [31]. Some of the major metabolites of imidacloprid are equally neurotoxic, acting on the same receptors, and are also persistent [41].

Three environmental risk limits for surface water are currently in use in the Netherlands. These are technical-scientific advisory values for achieving environmental quality standards.

The MTR stands for Maximum Permissible Risk (Dutch: Maximaal Toelaatbaar Risico), and is the environmental concentration at which the species in an ecosystem are considered safe from effects caused by the substance, based on as many toxicity studies as possible. The MTR imidacloprid was 13 ng l^−1^ at the time the data used in this study were collected [42]. In the context of the European Water Framework Directive a Maximum Permissible Concentration (MPC) has been derived, which is the concentration at which aquatic ecosystems and humans should be protected from effects due to long-term exposure. The MPC_eco,water_ for fresh water, based on ecotoxicological data for direct exposure, is set at 67 ng l^−1^ [43]. The Maximum Acceptable Concentration (MAC) is the concentration at which aquatic ecosystems should be protected from effects due to short-term exposure or concentration peaks. The MAC_eco,water_ for fresh water, based on ecotoxicological data for direct exposure, is set at 200 ng l^−1^ [43].

As one of the most-used insecticides the Netherlands, imidacloprid came highest in a ranking of substances that exceeded the MTR in 2004 [44]. It has been in the top 3 of that list every year since 2004 and number 1 in most years. The MTR for imidacloprid has been exceeded in almost half of all 9037 water samples in our dataset; the highest exceedance, measured in 2005 near Noordwijkerhout, was 320 µg l^−1^ [42] – this is almost 25,000 times the MTR, and about 56 times the 96-h LC_50_ for *Chironomus tentans* of 5.75 µg l^−1^ [10]. It is also well within the acute toxicity (48-h EC_50_) range (289–841.2 µg l^−1^) of the cladoceran *Ceriodaphna dubia* [45]. Imidacloprid norm exceeding is not exclusive to the Netherlands. Almost one fifth of water samples taken in California, USA exceeded the United States Environmental Protection Agency’s (EPA) Aquatic Life Benchmarks of 35 µg l^−1^ (acute) and 1.05 µg l^−1^ (chronic) for invertebrates and the concentrations found there also often exceeded European and Canadian toxicity directives [46].

Much research has already been conducted on the influence of neonicotinoid insecticides on various species under controlled conditions in the lab and in mesocosms. Here, we combined eight years of Dutch monitoring data on imidacloprid in surface water with eight years of monitoring data on macrofauna abundance to look at this influence on a nationwide scale, something that had not been done before. We combined 680,147 species abundance measurements [*x, y, date, species, abundance*] at 7380 unique locations *[x,y]* with 9037 imidacloprid concentration measurements [*x, y, date, concentration*] at 801 locations. Locations and dates differed across both datasets. To combine the datasets we used ≤1 km distance and ≤160 days time difference as criteria for coupling the abundance data to the concentration data (see Methods section for details). This resulted in a combined dataset of 18,898 records [*concentration, abundance, species*] for the years 1998 and 2003–2009. We analysed this dataset to answer the question: is there a relationship between neonicotinoid residues in the surface water, and the number of observed individuals per non-target species, in the Netherlands? Note that our approach of statistical analysis of observational data implies that even if we find a correlation, this does not necessarily imply causality, because there could be other factors that could be the main driver of the observed patterns of abundance. In the discussion we will reflect on this issue in more detail.

## Materials and Methods

### Data Collection

Data on imidacloprid concentrations in surface water in the Netherlands were obtained from the Dutch pesticides atlas [42]. This is a database with nationwide results from routine monitoring of pesticide residues in Dutch surface water covering almost 700 pesticides and metabolites. The monitoring program is effectuated by the Dutch water boards, Leiden University and the Board for the Authorisation of Plant Protection Products and Biocides (Ctgb), and at the time we obtained the data, they were available for the years 1998, and 2003 to 2009.

For all samples in which no imidacloprid could be detected or quantified, the dataset reports the limit of reporting (LOR) instead. These numbers are flagged in the dataset to alert the user that they do not represent the measured concentration but the LOR. This is because the real imidacloprid concentration in samples that tested negative for imidacloprid can be anything within the range of 0 to the LOR of the particular measurement method used. The values of the reporting limits vary across water boards and across years; in the dataset, LORs ranged from 5 ng l^−1^ to 190 ng l^−1^. Of those samples for which no true imidacloprid concentration was actually reported, we only included samples with LOR ≤7 ng l^−1^, because we were interested in the effects of low imidacloprid concentrations. In these cases we used the reporting limit as the imidacloprid concentration for these samples.

Initially, data on the distribution and abundance of aquatic macro-invertebrate species in Dutch surface water were obtained from Limnodata Neerlandica (www.limnodata.nl), an online database developed and maintained by the Dutch Foundation for Applied Water Research (STOWA) and containing data provided by the water boards, the Provinces and Rijkswaterstaat. These data were used in an earlier study by Van Dijk [47]. However, Verdonschot and Van Oosten-Siedlecka [48] showed that the majority of the data in the Limnodata database were not copied properly from the original datasets, and therefore might not be reliable. Therefore, we requested the original macro-invertebrate datasets directly from the water boards, and received files from 23 of the total 26. We did not succeed in getting in contact with the very small water board Blija Buitendijks. The water boards Noorderzijlvest and Reest en Wieden did not supply data. We received data for various years, but could only use those for 1998 and 2003 to 2009 because of the limitations in the imidacloprid dataset; for the year 2009 we used the data from January to June.

The data files we received from the 23 water boards did not all have the same layout. We applied several operations (see text S1) to standardize the data and make them suitable for our analysis. The water boards collect these data by taking water samples at a fixed set of locations in the Netherlands, and from those samples the aquatic macro-invertebrate species and their abundance are determined. This means that all macro-invertebrate species found have at least one aquatic life stage. A standardized macro fauna net is used, with opening 0.30×0.20 m, depth 0.5 m and mesh size 0.5 mm. For each sample the standard net is moved through the water over a length of 5 m. Species in the samples are determined and individuals per species are counted. Only species present in the sample are reported, which implies that the minimum abundance of each species in each sample in the dataset is 1 and not 0. A detailed description of the sampling methods can be found in [49]. The definition of aquatic macro-invertebrate species is based on two criteria: the size of the representatives per taxonomic group (chiefly >0.5 mm), and the ease with which the taxonomic groups can be determined using common sampling methods.

### Pairing Macro-invertebrate Data with Imidacloprid Data

The locations of the measurements of the imidacloprid concentrations and those of the samples of aquatic macro-invertebrates were mostly different. Chemical and biological samples were situated at various distances from each other. The same is true for the dates on which the measurements and samples were taken. To be able to investigate the relationship between imidacloprid concentration and species abundance, we paired the two datasets by making a selection based on a limited distance between the measurement location and sampling location and a small difference in dates between the measurement and sampling. For each macro-invertebrate sample we paired the data with the imidacloprid measurements located within a radius of 1 km, and no more than 160 days difference (one way). When more than one imidacloprid measurement was found that met these criteria, the median imidacloprid concentration of these measurements was used. The period of 160 days was based on the high end of the range of reported half-life times of imidacloprid in water [36], [40]. Further, using the 160 days time window for our analysis allows for chronic and sublethal effects on population and reproduction to take effect, which would otherwise be overlooked in the analysis. In contrast to other pesticides, where recovery can occur after pulse exposure, aquatic invertebrate communities exposed to imidacloprid and other neonicotinoids take a long time to recover for two reasons: either the populations exposed die completely through chronic exposure, or they are unable to reproduce due to chronic weakness.

### Statistical Analysis

All years and all places were pooled into one data set because we are mainly interested in the overall link between imidacloprid concentration and macro-fauna abundance and not in spatial temporal patterns. We tested the data for spatial autocorrelation using variograms exploring distances between samples up to 10 km. For the further analysis we used various ways of aggregating the data for different species: all species pooled, species pooled per order (e.g. all Diptera pooled) and non-aggregated (analysis at species level). First, scatter plots were made to investigate the dependence of species abundance on imidacloprid concentration. Because of the skewed distribution of the data a log_10_-transformation was performed on the abundance data and imidacloprid concentration data. To enable easy comparison between species, a linear regression analysis was carried out on the log-transformed data. This is an over simplified metric for the strength of association but it enables an ordinal ranking of species according to strength of association. The significance of the regression coefficients was then tested with an Analysis of Variance (ANOVA).

Next, we reverted to an approach with a higher statistical power: a nonparametric test was performed to test the significance of the differences between the species abundance at imidacloprid concentrations above and below a water quality norm for imidacloprid (MTR, MPC, MAC). Because of the non-normal distribution of the non-transformed data on abundance, Mann-Whitney *U* tests were carried out to test the significance of differences in average abundance between the pooled samples above and the pooled samples below each water quality norm. Differences were considered significant at *P*<0.05. All datasets were analysed with the statistical package SPSS 16.0 for Windows (SPSS Inc., Chicago, Illinois, USA).

## Results

### Relationship between Imidacloprid Concentration and Species Abundance

**Figure 1:**
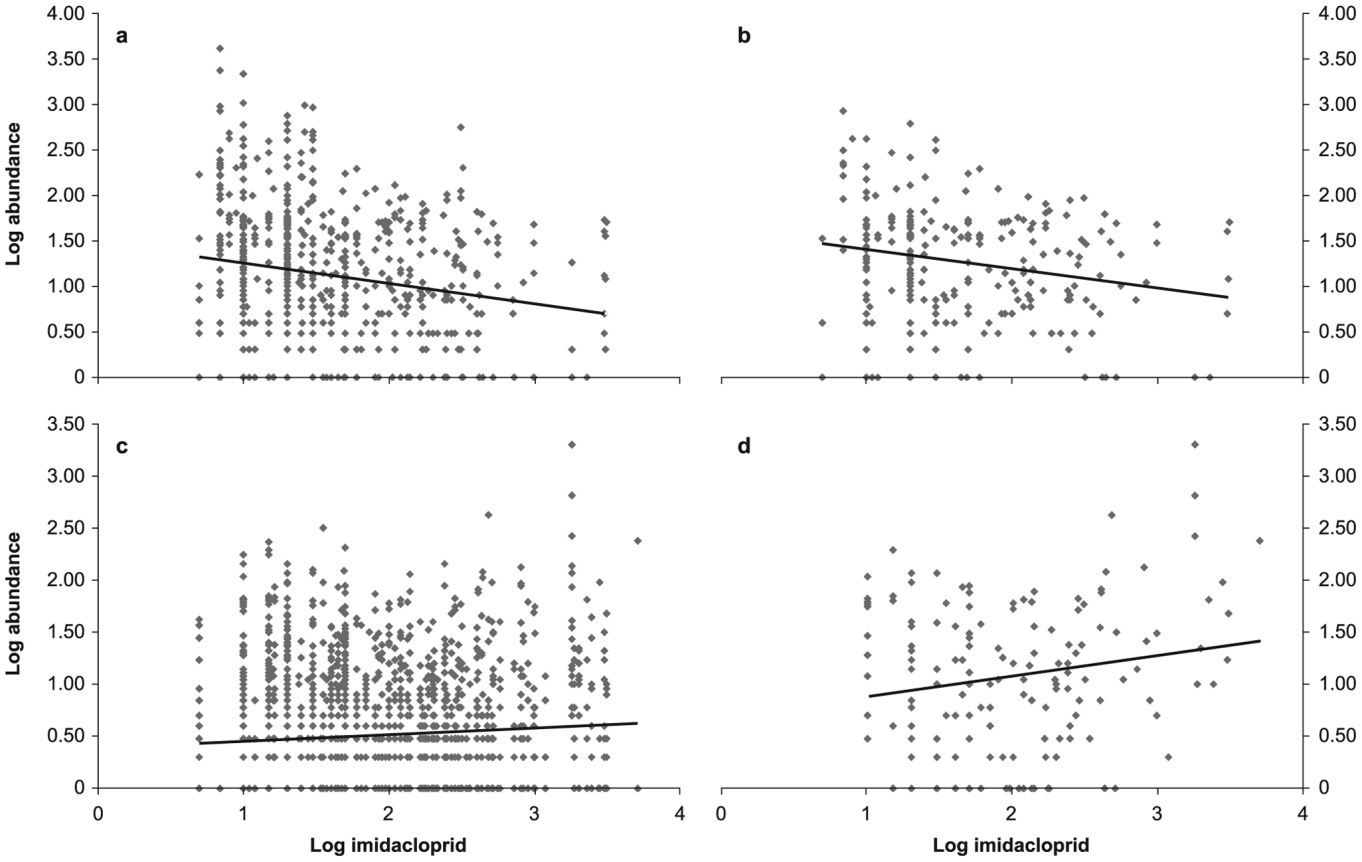
Relationship between log_10_ imidacloprid concentration and log_10_ Amphipoda and Actinedida abundance in surface water. a) Amphipoda (*P* = <0.001), b) its most abundant species *Gammarus tigrinus* (*P* = 0.001), c) Actinedida (*P* = <0.001), d) its most abundant species *Limnesia undulata* (*P* = 0.022).

We did not find any spatial auto correlation in the abundance data. In the imidacloprid concentrations we found spatial autocorrelation for the short distances, but only between data points situated less than 3 km apart. Visual inspection of the scatter plots of abundance versus imidacloprid concentration (figures 1 and S1–S6) clearly show that at high imidacloprid concentrations, high abundance is rare while at low concentrations it is common. The simplified linear regression shows a significant negative relationship between species abundance and imidacloprid concentration for all species pooled, as well as for the separate orders Amphipoda (crustaceans), Diptera (true flies), Ephemeroptera (mayflies), Isopoda (custaceans) and Basommatophora (snails). For these orders the species abundance decreased significantly with increasing imidacloprid concentration (figure 1, table 1; figures S1–S6). The strongest decrease in species abundance was found for Amphipoda, with a slope of regression line *β* = −0.180 and *P*<0.001, and Ephemeroptera (*β* = −0.157, *P* = 0.001). For each of the five orders mentioned above, one of the three most abundant species in the sampling data showed a significant negative relationship as well. Most of the other abundant species in these orders also showed a negative tendency, but those relationships were not significant at *P*<0.05. The negative relationship for the order Odonata (dragonflies and damselflies) was nearly significant.

**Table 1:** Results of regression analysis on the relationship between imidacloprid concentration and species abundance for all macro-invertebrate orders together, for orders with a total species abundance n >300, and for the three most abundant species of each order.

Order	Species	*F*	*β*	*n*	*P*	*r* ^2^
All orders		71.863	−0.062	18898	<0.001 *	0.004
Amphipoda		21.733	−0.180	652	<0.001 *	0.032
	*Gammarus duebeni*	3.966	−0.364	28	0.057	0.132
	*Gammarus tigrinus*	10.984	−0.206	249	0.001 *	0.043
	*Gammarus zaddachi*	0.848	−0.257	14	0.375	0.060
Actinedida		12.206	0.075	2148	<0.001 *	0.006
	*Arrenurus sinuator*	0.516	0.062	134	0.474	0.004
	*Limnesia undulata*	5.373	0.185	153	0.022 *	0.034
	*Unionicola crassipes*	0.365	−0.058	112	0.547	0.003
Basommatophora		12.649	−0.086	1684	<0.001 *	0.007
	*Gyraulus albus*	5.410	−0.172	179	0.021 *	0.030
	*Hippeutis complanatus*	3.635	−0.181	109	0.059	0.033
	*Physella acuta*	2.523	−0.127	155	0.114	0.16
Coleoptera		0.435	0.018	1379	0.510	<0.001
	*Haliplus fluviatilis*	0.777	0.110	66	0.381	0.012
	*Noterus clavicornis*	0.145	0.041	86	0.705	0.002
	*Noterus crassicornis*	0.100	0.039	68	0.752	0.002
Diptera		25.799	−0.073	4757	<0.001 *	0.005
	*Endochironomus albipennis*	2.296	−0.101	227	0.131	0.010
	*Glyptotendipes pallens*	13.452	−0.434	60	0.001 *	0.188
	*Polypedilum nubeculosum*	7.122	0.187	198	0.008 *	0.035
Ephemeroptera		11.926	−0.157	471	0.001 *	0.025
	*Caenis horaria*	9.170	−0.352	67	0.004 *	0.124
	*Caenis robusta*	3.149	−0.174	103	0.079	0.030
	*Cloeon dipterum*	1.882	−0.098	197	0.172	0.010
Hemiptera		2.490	−0.040	1583	0.115	0.002
	*Micronecta scholtzi*	0.252	0.048	111	0.617	0.002
	*Plea minutissima*	0.448	−0.085	64	0.506	0.007
	*Sigara striata*	0.231	−0.031	246	0.631	0.001
Isopoda		5.127	−0.102	493	0.024 *	0.010
	*Asellus aquaticus*	0.011	−0.007	247	0.915	<0.001
	*Proasellus coxalis*	5.142	−0.210	114	0.025 *	0.044
	*Sphaeroma hookeri*	1.292	−0.252	21	0.270	0.064
Neotaenioglossa		0.260	−0.240	450	0.610	0.001
	*Bithynia leachi*	0.481	0.065	114	0.489	0.004
	*Bithynia tentaculata*	3.530	0.132	202	0.062	0.017
	*Potamopyrgus antipodarum*	7.155	−0.276	89	0.009 *	0.076
Odonata		3.817	−0.079	604	0.051 *	0.006
	*Erythromma najas*	0.480	−0.143	25	0.495	0.020
	*Erythromma viridulum*	0.594	−0.144	30	0.447	0.021
	*Ischnura elegans*	6.164	−0.175	197	0.014 *	0.031
Rhynchobdellae		0.006	−0.003	924	0.937	<0.001
	*Alboglossiphonia heteroclita*	0.169	−0.042	100	0.682	0.002
	*Helobdella stagnalis*	0.598	0.053	215	0.440	0.003
	*Theromyzon tessulatum*	0.455	−0.088	61	0.502	0.008
Trichoptera		0.157	−0.019	447	0.692	<0.001
	*Mystacides longicornis*	0.208	−0.071	43	0.651	0.005
	*Oecetis lacustris*	7.118	−0.397	40	0.011 *	0.158
	*Triaenodes bicolor*	0.461	0.127	30	0.503	0.016
Tubificidae		1.570	−0.035	1254	0.210	0.001
	*Ophidonais serpentina*	0.029	−0.018	89	0.865	<0.001
	*Stylaria lacustris*	0.873	−0.075	157	0.351	0.006
	*Tubifex costatus*	0.008	−0.032	10	0.930	0.001
Veneroida		0.081	−0.012	591	0.776	<0.001
	*Dreissena polymorpha*	0.014	−0.019	41	0.906	<0.001
	*Pisidium nitidum*	0.313	−0.068	69	0.578	0.005
	*Sphaerium corneum*	0.023	0.020	58	0.881	<0.001

* Indicates a significant relationship at *P*<0.05. *F* is the *F* ratio, *β* is the slope of the regression line. The data are log transformed so the numbers are dimensionless.

For the order Actinedida (water mites), a reverse trend was observed. Here, a significant positive relationship was found, which means that species abundance for this order increases when the imidacloprid concentration in surface water increases. This was also found for the Actinedida species *Limnesia undulata*. *Polypedilum nubeculosum,* a species of Diptera, also showed a positive relationship (*β* = 0.187, *P* = 0.008), while *Glyptotendipes pallens*, the most abundant Diptera species in the water samples, had a significant negative relationship (*β* = −0.434, *P* = 0.001). For the orders Neotaenioglossa (sea snails) and Trichoptera (caddisflies), one of the three most abundant species showed a significant negative relationship as well. The *F* ratio in table 1 indicates the ratio of the explained variance over the unexplained variance. The *r* ^2^ values in table 1 show that the oversimplified linear regression model leaves the major part of the variability unexplained. Note that we pooled all data irrespective of the time of the year of sampling, this means that the seasonal cycles in abundance may account for a substantial part of the variability for many species.

### Water Quality Norms and Aquatic Macro-invertebrate Abundance

**Figure 2:**
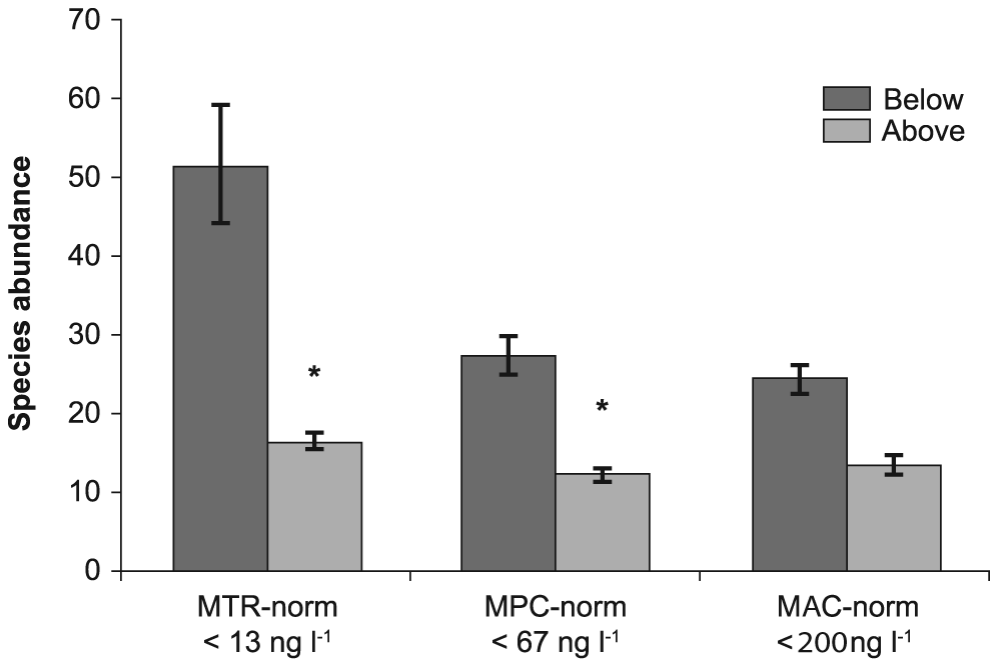
Macro-invertebrate abundance in surface water samples below and above Dutch imidacloprid norms for surface water. Mean and standard error of abundance is shown. We used median imidacloprid concentrations. Dependent variables were tested separately using the Mann-Whitney test. *Indicates significant differences at *P*<0.05. MTR = Maximum Permissible Risk imidacloprid, MPC = Maximum Permissible Concentration imidacloprid, MAC = Maximum Acceptable Concentration imidacloprid (see text).

The three environmental risk limits used in the Netherlands to help achieve environmental quality are not met in many parts of the country [42]. This may influence species abundance in the surface water. Figure 2 shows the mean species abundance above and below the environmental risk limits for all species pooled. Clear and significant differences were found between species abundance below and above the limits of two water quality norms. The strictest norm, the MTR of 13 ng l^−1^ imidacloprid in surface water, showed the highest difference in average species abundance: a 3-fold difference (Mann-Whitney *U* test: *P*<0.001). The less strict MPC-norm, of 67 ng l^−1^ imidacloprid, also showed a significant difference in species abundance below and above the limit (*P*<0.001), but here the difference was smaller: a 2-fold difference. The MAC-norm of 200 ng l^−1^ imidacloprid in surface water, which is about 15 times less strict than the MTR-norm, showed a smaller difference in species abundance which was not significant (*P* = 0.065).

## Discussion

Visual inspection of the scatter plots convincingly shows that at high imidacloprid concentrations, high macro-fauna abundance is rare in comparison to high abundance at low imidacloprid concentrations. The simplified regression analysis showed a significant negative relationship between imidacloprid concentration and macro-invertebrate abundance. Such an association does not necessarily imply that imidacloprid is the main cause for lower species abundance, as there can be other factors and confounders that play a role in the observed patterns of abundance. In 1965 Sir Austin Bradford Hill [50] introduced nine criteria for distinguishing between a chance association and a true cause and effect: 1. strength of association, 2. consistency, 3. specificity, 4. temporality, 5. biological gradient, 6. biological plausibility, 7. biological coherence, 8. experimental evidence, and 9. analogy. These criteria are widely used by epidemiologists nowadays [51], [52]. Their usefulness for the scientific inquiry on causal links, and for the justification of policy intervention based on the available evidence, has been widely recognized [53]. We will briefly discuss how the link between imidacloprid and reduced species abundance scores on these criteria.

Firstly, our statistical analysis shows a high strength of association with a high significance. The second criterion, consistency, also scores high; in our dataset we made a few random subsets of our data and found that the correlation (for all species pooled) is not sensitive to the years that we include in the analysis nor to the areas that we include: the pattern is consistent across time and space. Regarding the third criterion, specificity, the score is low because there are many potential factors that could reduce species abundance. However, the reason why we focussed our analysis on imidacloprid is that since 2004, it has been the insecticide with the highest number of samples that exceed the Dutch aquatic toxicity norm for surface water. On average, about half of all samples from the years 2004 to present in the nation-wide monitoring program violates this standard. Further, in these samples, the distance to the norm is extreme compared to other agrochemicals in the same surface waters. For that reason imidacloprid is a prime suspect compared to other pesticides. Also, we tested for spatial autocorrelation in the abundance data, both in the untransformed and in the log-transformed data, and did not find any autocorrelation (data not shown here). Consequently we have no reason to assume that landscape quality would be a major confounder in our case, but we cannot completely exclude it either. The fourth criterion scores high as in combining the datasets from biological and chemical sampling the date of the chemical sample is always before or at the date of the biological sample. We specifically used a range of 0 to 160 days for the (one way) time difference between the biological sampling and the chemical sampling, which is long enough for sublethal and chronic mechanisms to induce effects at population and community levels. For criterion 5, biological gradient, it is obvious from the data plots and the regression analysis that increased exposure to imidacloprid is associated with an increased effect. There clearly is a biological gradient, so this criterion also scores high. As regards criterion 6, the present day knowledge on sublethal effects of neonicotinoids on invertebrate reproduction adds to the biological plausibility that imidacloprid is indeed the main causal factor. On top of that, recent insights on the chronic toxicity profile of neonicotinoids, in particular the notion that the toxicity is reinforced by exposure time [24], implies that even the lowest concentrations, when sustained over a long period, will negatively impact invertebrates. Criterion 7, biological coherence, also scores high. Our study is consistent with a wide range of earlier studies as we will discuss further on in this section. The link between abundance and imidacloprid also scores high on criterion 8, experimental evidence. A large number of laboratory studies and mesocosm expriments discussed earlier in this paper all confirm the high toxicity of imidacloprid on invertebrates and clearly indicate community effects. Finally, the link also scores high on criterion 9, analogy, because for other neonicotinoids such as thiacloprid similar strong effects on community level have been observed in mesocosms (e.g. [54]).

While we still cannot exclude that our analysis overlooked confounders, the application of the causality criteria provides strong grounds to believe that the link between imidacloprid and abundance is indeed causal. Still, it remains advisable to further investigate whether a multivariate regression analysis, using a wider range of suspect chemicals still pinpoints imidacloprid as the main suspect, but the present data availability limits the statistical power of such a multivariate analysis, making extension of the systematic chemical and biological monitoring programs of surface water advisable as well.

Our findings are consistent with many other studies (see references in [47]) which reported a negative impact of neonicotinoid insecticides on a high number of non-target species. Flying insects appeared to be the most vulnerable to neonicotinoids in these studies [10], [23], [55]–[57]. In this study, the vulnerability to neonicotinoids of flying instects with an aquatic larval stage was also demonstrated: a significant negative relationship was found for the orders Diptera and Ephemeroptera, and a nearly significant relationship for Odonata (table 1). The caddisfly *Oecetis lacustris* of the order Trichoptera showed a strong negative relationship as well. Trichoptera are widely used in water quality assessments [58]–[60] and a high species richness of this order is generally assumed to indicate a good water quality. The strong decline we found for *Oecetis lacustris* at locations with higher imidacloprid concentrations can be seen as an indication that imidacloprid is an important factor reducing water quality.

With our approach we found effects at lower concentrations than known from mesocosm studies. A possible explanation is that mesocosm studies may underestimate the long term effects because the recovery observed in mesocosm studies is probably due to re-colonization by external individuals, not by recovery of the individuals affected by the exposure.

A reverse effect was found for the order Actinedida: our regression analysis showed a significant positive relationship between imidacloprid concentration and Actinedida abundance. This is consistent with the results of Szczepaniec et al. [61] who found spider mite outbreaks after the use of imidacloprid on trees. The outbreaks were probably caused by a positive effect of imidacloprid on mite reproduction by increasing the hatch rate [62]. However, positive relationships are exceptional in the case of imidacloprid (see table 1).

Besides the direct negative effects found on species living in the water, indirect effects of imidacloprid on the food chain can be expected as well. Experiments in imidacloprid-treated rice fields by Hayasaka et al. [63] showed direct negative effects on the species abundance of the zooplankton community, leading to the indirect effect of growth suppression in the fish feeding on the zooplankton species. Sanchez-Bayo and Goka [64] found indirect effects on algae growth in rice fields, after changes of the arthropod communities induced by imidacloprid. Indirect effects of the neoniconinoid thiacloprid on the food chain and ecosystem functions were also observed by Englert et al. [65] in a study on predator-prey interactions of gammarids and mayflies. Increased thiacloprid concentrations in surface water increased predation by *Gammarus fossarum* (Amphipoda) on *Baetis rhodani* (Ephemeroptera) nymphs, probably because of the impairment by thiacloprid of the predator avoidance behavior of *B. rhodani*. With the increased consumption of *B. rhodani* nymphs by *G. fossarum*, a reduction was observed in leaf consumption by *G. fossarum,* which can be explained by the preference of *G. fossarum* for food of high nutritional value. This reduced leaf consumption may translate into impairment of the ecosystem function of leaf litter breakdown. Other studies on aquatic decomposer organisms [11], [66] also showed significant adverse effects (feeding inhibition) of imidacloprid on aquatic insects and high mortality. Antipredator responses to imidacloprid exposure were found by Pestana et al. [67] in the zooplanktonin grazer *Daphnia magna*.

Even at low levels of toxicants community-level effects can be found, as was shown in another study [54]. We suggest that not only organism-level effects should be considered for environmental risk assessment of insecticides, but community-level effects as well.

Leaf decomposition by leaf-shredding insects was found to be significantly reduced. Cumulative ecological impacts of insecticides were shown in experiments in rice fields with two successive annual treatments of imidacloprid and fipronil [63]. The abundance of aquatic organisms during both years was significantly lower in both insecticide-treated fields compared to the control, and large changes in aquatic community composition were observed. These results show that the impacts of insecticides cannot be accurately assessed during short-term monitoring studies. Like Wijngaarden et al. [68] suggested, we too recommend that the long-term ecological risks of their residues are included in an assessment of insecticide effects at the community level.

Besides cumulative effects, imidacloprid is also known to act synergistically with other chemicals. For instance, eight days’ exposure to a mixture of the nonylphenol polyethoxylate, R-11 and imidacloprid resulted in a population size which was three times smaller than with R-11 alone, and 13 times smaller than with imidacloprid alone in the crustacean *Ceriodaphnia dubia* [69]. The 96-h LC_50_ for imidacloprid in the presence of atrazine was significantly lower compared to imidacloprid alone for the daggerblade grass shrimp *Palaemonetes pugio* [70]. In the work of Loureiro et al. [71] on synergistic effects on *Daphnia magna*, synergism was observed for acute exposures of imidacloprid and thiacloprid mixtures (immobilization), and antagonism for feeding rates at sublethal concentrations. For imidacloprid and chlorpyrifos, antagonism was found in both exposures. In another study three widely used synergists were tested: piperonyl butoxide, triphenyl phosphate, and diethyl maleate. All tested synergists significantly amplified the toxic effect of imidacloprid on the wasp *Diaeretiella rapae*, piperonyl butoxide having the greatest impact [72]. Piperonyl butoxide, triflumizole and propiconazole increase the toxicity to honey bees of imidacloprid 1.70-, 1.85- and 1.52-fold respectively [73]. These substances are putative inhibitors of cytochrome P450s, a group of enzymes involved in the detoxification of xenobiotics such as pesticides, which explains their synergistic action.

Neonicotinoids have cumulative effects with exposure time [26], which become relevant for aquatic organisms which are constantly exposed to low levels of many contaminants. While most pesticides do not have toxic effects below a certain level (NOEC or NOEL), the cumulative effects of neonicotinoids imply that even the lowest concentrations have toxic effects if sustained over a long period, which is especially relevant for species with a long life span or a long aquatic stage [57].

Our results show that aquatic macro-invertebrates in Dutch surface water are less abundant at locations with higher imidacloprid concentrations. This provides reason for concern because the three water quality standards applied in the Netherlands to achieve ecological protection are not met in many parts of the country [42], and especially in agricultural areas with greenhouses and crops like bulbs, where concentrations up to hundreds of µg l^−1^ imidacloprid are being found in the surface water.

Our results further show that – of the existing norms - the strictest norm, the MTR of 13 ng l^−1^ imidacloprid in surface water, makes the greatest difference for species abundance and is thus the only existing norm that could protect aquatic ecosystems. We cannot exclude that a norm lower than the best current Limit of Reporting of the measurement methods for imidacloprid concentration would even be more effective in protecting aquatic life. For the much less strict MAC-norm of 200 ng l^−1^, there is no significant difference in average species abundance between the samples from locations where the norm is met and those where the norm is exceeded. It follows from the comparison of protectiveness of the various norms (figure 2) that a major drop in macro fauna abundance occurs when concentrations go up from exceeding 13 ng l^−1^ to exceeding 67 ng l^−1^. Our findings imply that the MTR-norm of 13 ng l^−1^ seems more like a lowest effect concentration. If adequate protection of aquatic ecosystems is the goal, a stricter norm should be set. If we take a safety factor of 10, a standard of 1 ng l^−1^ is recommendable. Note that this is below the detection limit of the imidacloprid measurement methods currently in use by the Dutch Water Boards.

While a large amount of evidence exists from laboratory single-species and mesocosm experiments, our study is the first large-scale research based on multiple years of actual field monitoring data that shows that neonicotinoid insecticide pollution occurring in surface water has a strong negative effect on aquatic invertebrate life, with potentially far-reaching consequences for the food chain and ecosystem functions. The combination of nation-wide monitoring data on insecticide concentrations and aquatic macro-invertebrates creates a valuable instrument for the analysis of the impacts of different pesticides and the evaluation of environmental policy. Given the fact that the world-wide use of neonicotinoid insecticides is still growing, and given its high leaching potential and its high persistence in water and soil, it is important to sustain and extend chemical monitoring schemes of surface water, and further analysis of the major impacts this pollution has on biodiversity and ecosystem services.

## Supporting Information

Figure S1 **Relationship between log_10_ imidacloprid concentration and log_10_ Basommatophora and Diptera abundance in surface water.** a) Basommatophora (*P*<0.001), b) its most abundant species *Gyraulus albus* (*P* = 0.021), c) Diptera (*P*<0.001), d) its most abundant species *Endochironomus albipennis* (*P* = 0.131). The first three relationships are significant at *P*<0.05.
(TIF)

Figure S2 **Relationship between log_10_ imidacloprid concentration and log_10_ Ephemeroptera and Isopoda abundance in surface water.** a) Ephemeroptera (*P* = 0.001), b) its most abundant species *Cloeon dipterum* (*P* = 0.172), c) Isopoda (*P* = 0.024), d) its most abundant species *Asellus aquaticus* (*P* = 0.915). The negative relationships for the orders are significant at *P*<0.05.
(TIF)

Figure S3 **Relationship between log_10_ imidacloprid concentration and log_10_ Coleoptera and Hemiptera species abundance in surface water.** a) Coleoptera (*P* = 0.510), b) its most abundant species *Noterus clavicornis* (*P* = 0.705), c) Hemiptera (*P* = 0.115), d) its most abundant species *Sigara striata* (*P* = 0.617).
(TIF)

Figure S4 **Relationship between log_10_ imidacloprid concentration and log_10_ Neotaenioglossa and Odonata abundance in surface water.** a) Neotaenioglossa (*P* = 0.610), b) its most abundant species *Bithynia tentaculata* (*P* = 0.062), c) Odonata (*P* = 0.051), d) its most abundant species *Ischnura elegans* (*P* = 0.014). The negative relationship for the order Odonata is nearly significant at *P*<0.05; the relationship for *Ischnura elegans* is significant.
(TIF)

Figure S5 **Relationship between log_10_ imidacloprid concentration and log_10_ Rhynchobdellae and Trichoptera abundance in surface water.** a) Rhynchobdellae (*P* = 0.937), b) its most abundant species *Helobdella stagnalis* (*P* = 0.440), c) Trichoptera (*P* = 0.692), d) its most abundant species *Mystacides longicornis* (*P* = 0.651).
(TIF)

Figure S6 **Relationship between log_10_ imidacloprid concentration and log_10_ Tubificidae and Veneroida abundance in surface water.** a) Tubificidae (*P* = 0.210), b) its most abundant species *Stylaria lacustris* (*P* = 0.351), c) Veneroida (*P* = 0.776), d) its most abundant species *Pisidium nitidum* (*P* = 0.578).
(TIF)

Text S1 **The dataset and operations performed on the raw dataset of abundance data.**
(DOC)
