# Supplementary figures and images for: Macro-Invertebrate Decline in Surface Water Polluted with Imidacloprid
Source: PLoS One. 2013 May 1;8(5):e62374. doi: 10.1371/journal.pone.0062374 (PMC3641074; doi:10.1371/journal.pone.0062374)

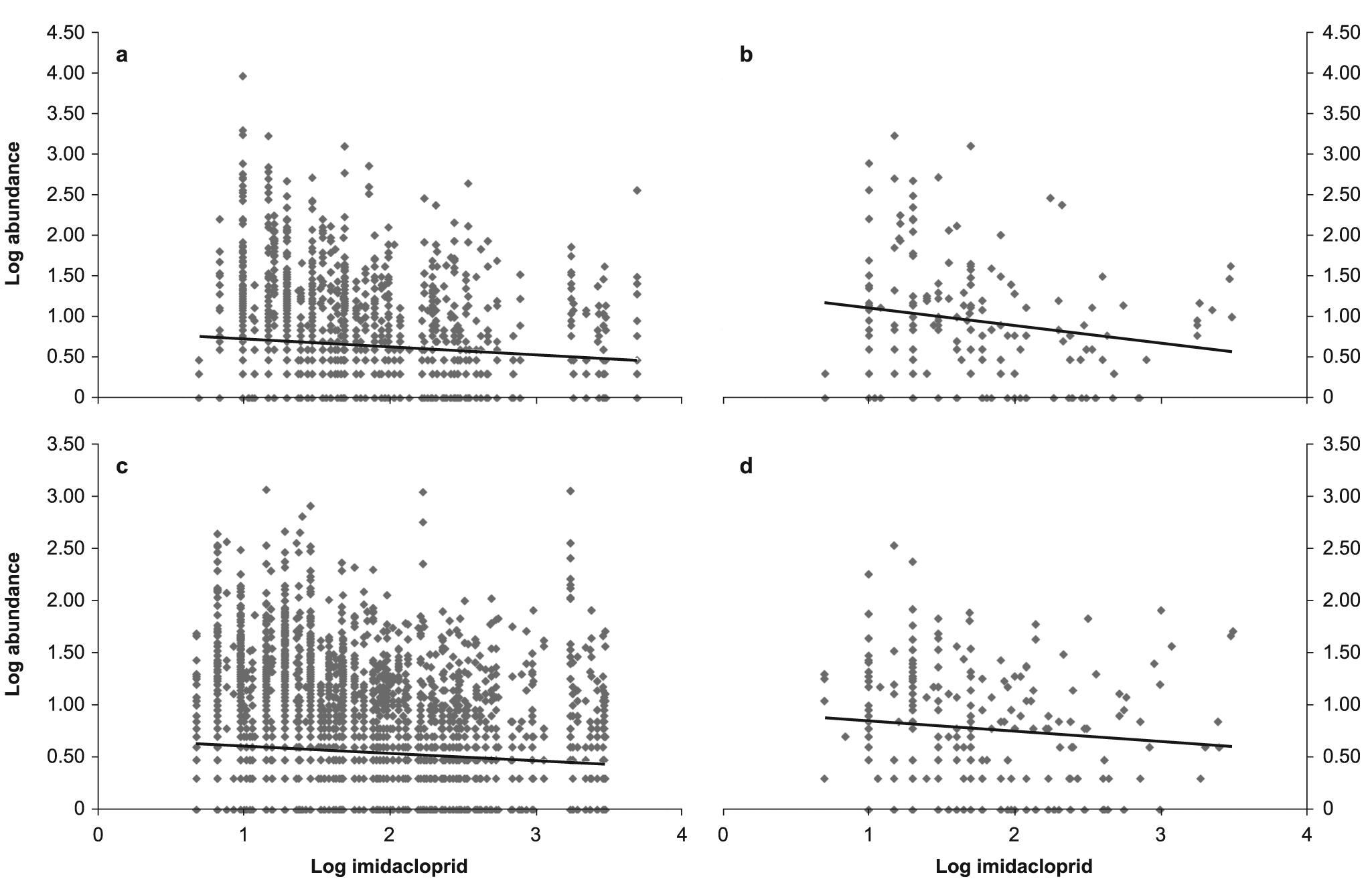

Supplement: Figure S1 — Relationship between log10 imidacloprid concentration and log10 Basommatophora and Diptera abundance in surface water. a) Basommatophora (P<0.001), b) its most abundant species Gyraulus albus (P = 0.021), c) Diptera (P<0.001), d) its most abundant species Endochironomus albipennis (P = 0.131). The first three relationships are significant at P<0.05. (TIF) [file pone.0062374.s001.tif]

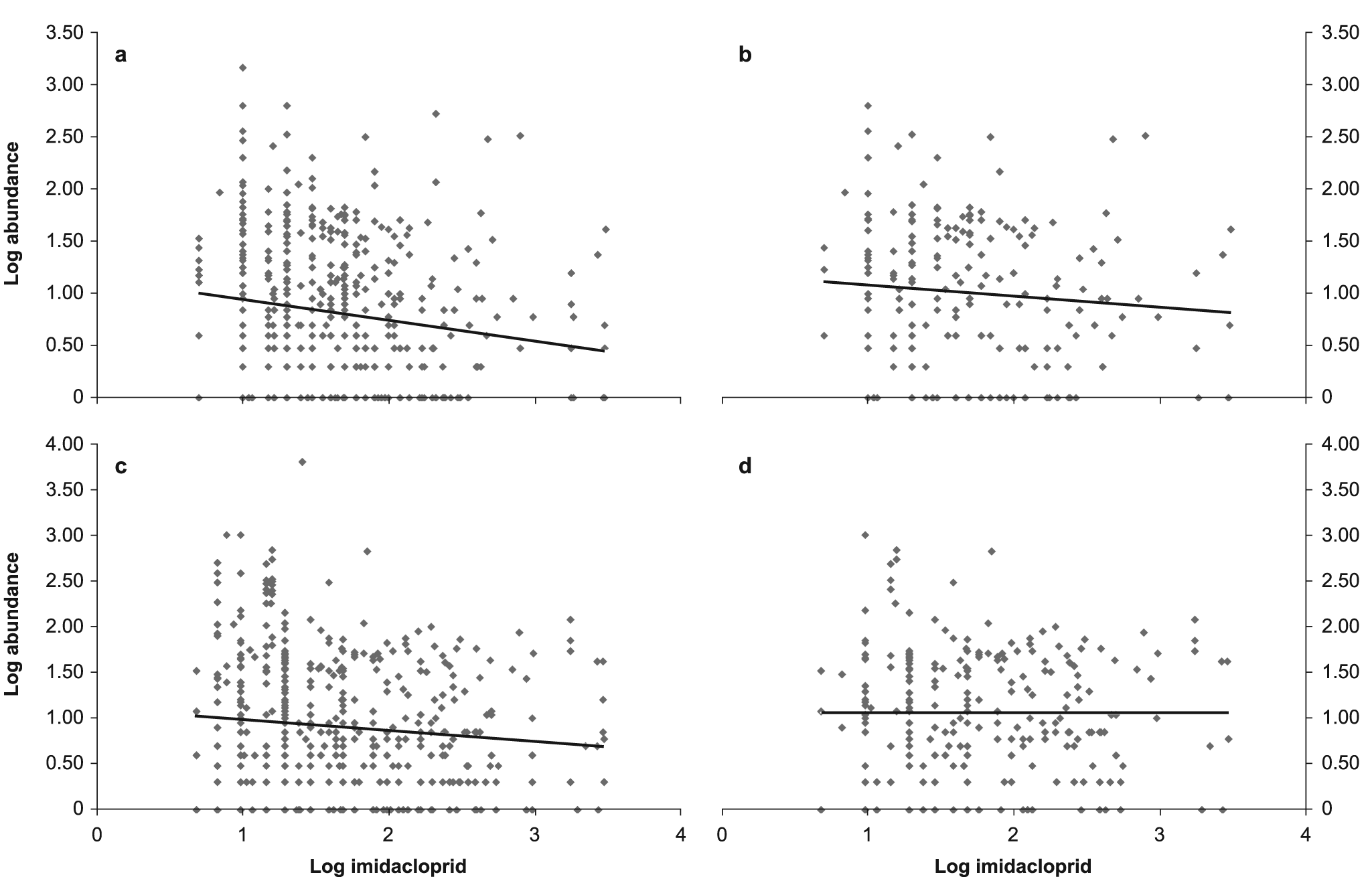

Supplement: Figure S2 — Relationship between log10 imidacloprid concentration and log10 Ephemeroptera and Isopoda abundance in surface water. a) Ephemeroptera (P = 0.001), b) its most abundant species Cloeon dipterum (P = 0.172), c) Isopoda (P = 0.024), d) its most abundant species Asellus aquaticus (P = 0.915). The negative relationships for the orders are significant at P<0.05. (TIF) [file pone.0062374.s002.tif]

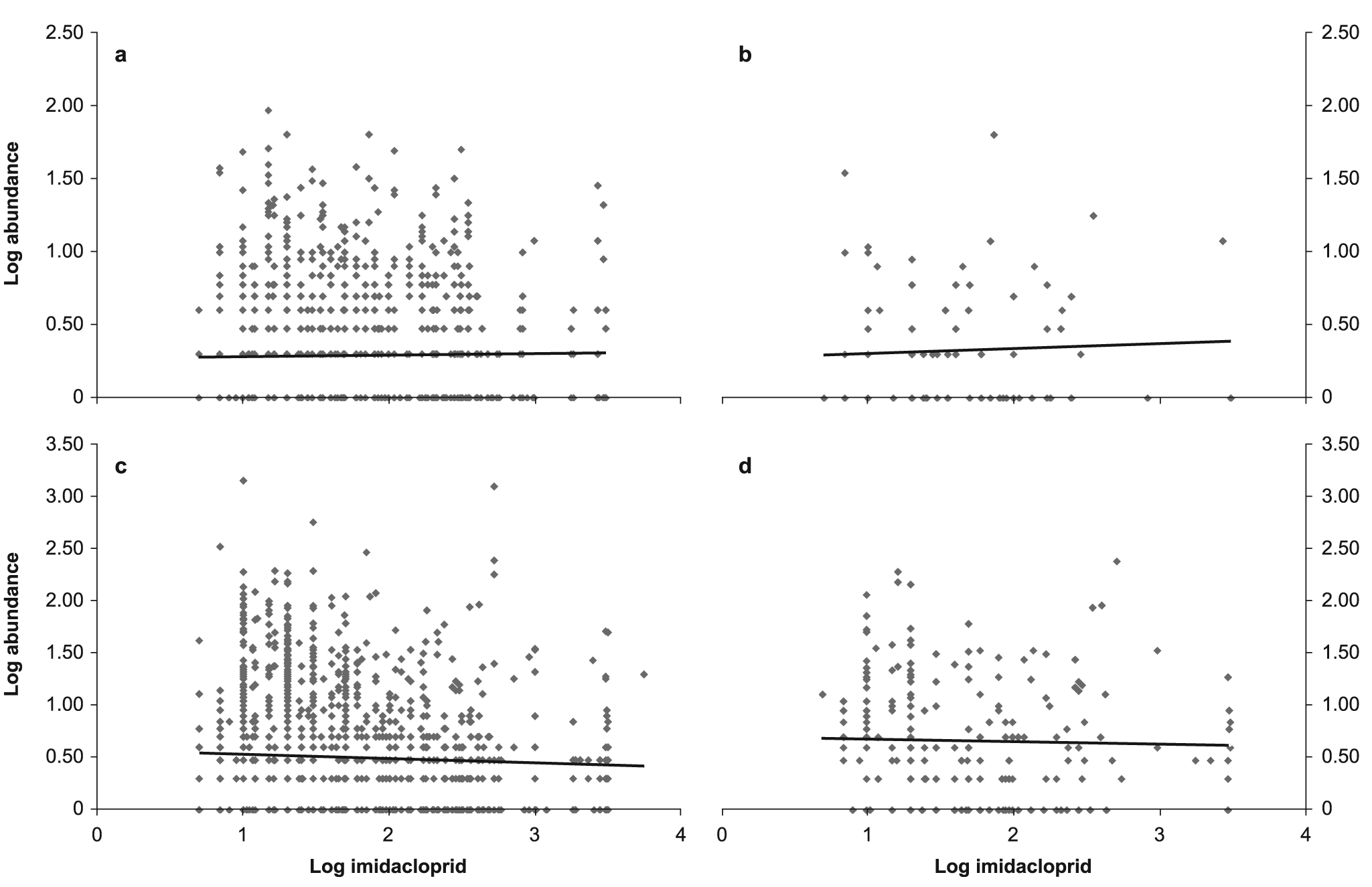

Supplement: Figure S3 — Relationship between log10 imidacloprid concentration and log10 Coleoptera and Hemiptera species abundance in surface water. a) Coleoptera (P = 0.510), b) its most abundant species Noterus clavicornis (P = 0.705), c) Hemiptera (P = 0.115), d) its most abundant species Sigara striata (P = 0.617). (TIF) [file pone.0062374.s003.tif]

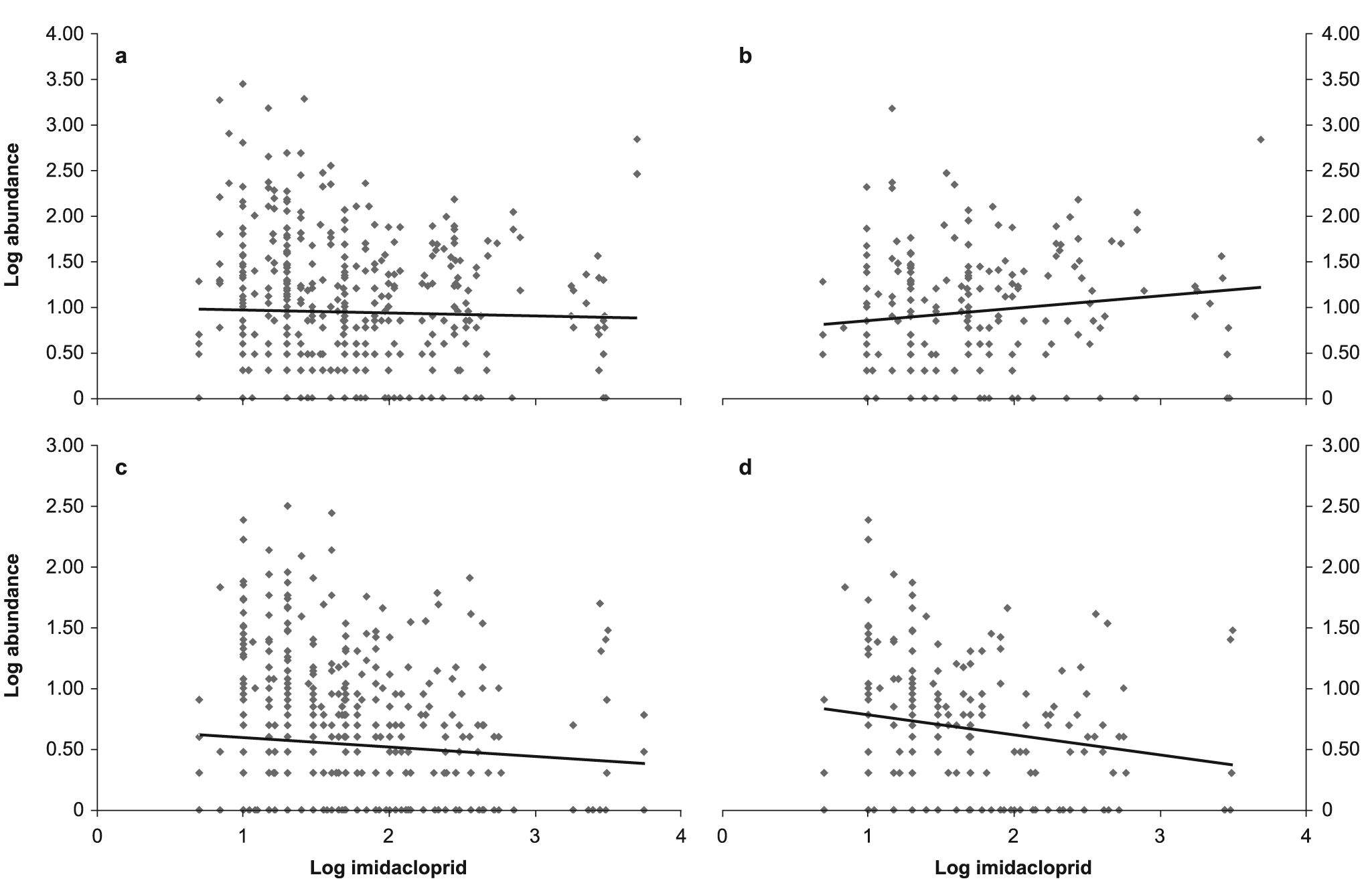

Supplement: Figure S4 — Relationship between log10 imidacloprid concentration and log10 Neotaenioglossa and Odonata abundance in surface water. a) Neotaenioglossa (P = 0.610), b) its most abundant species Bithynia tentaculata (P = 0.062), c) Odonata (P = 0.051), d) its most abundant species Ischnura elegans (P = 0.014). The negative relationship for the order Odonata is nearly significant at P<0.05; the relationship for Ischnura elegans is significant. (TIF) [file pone.0062374.s004.tif]

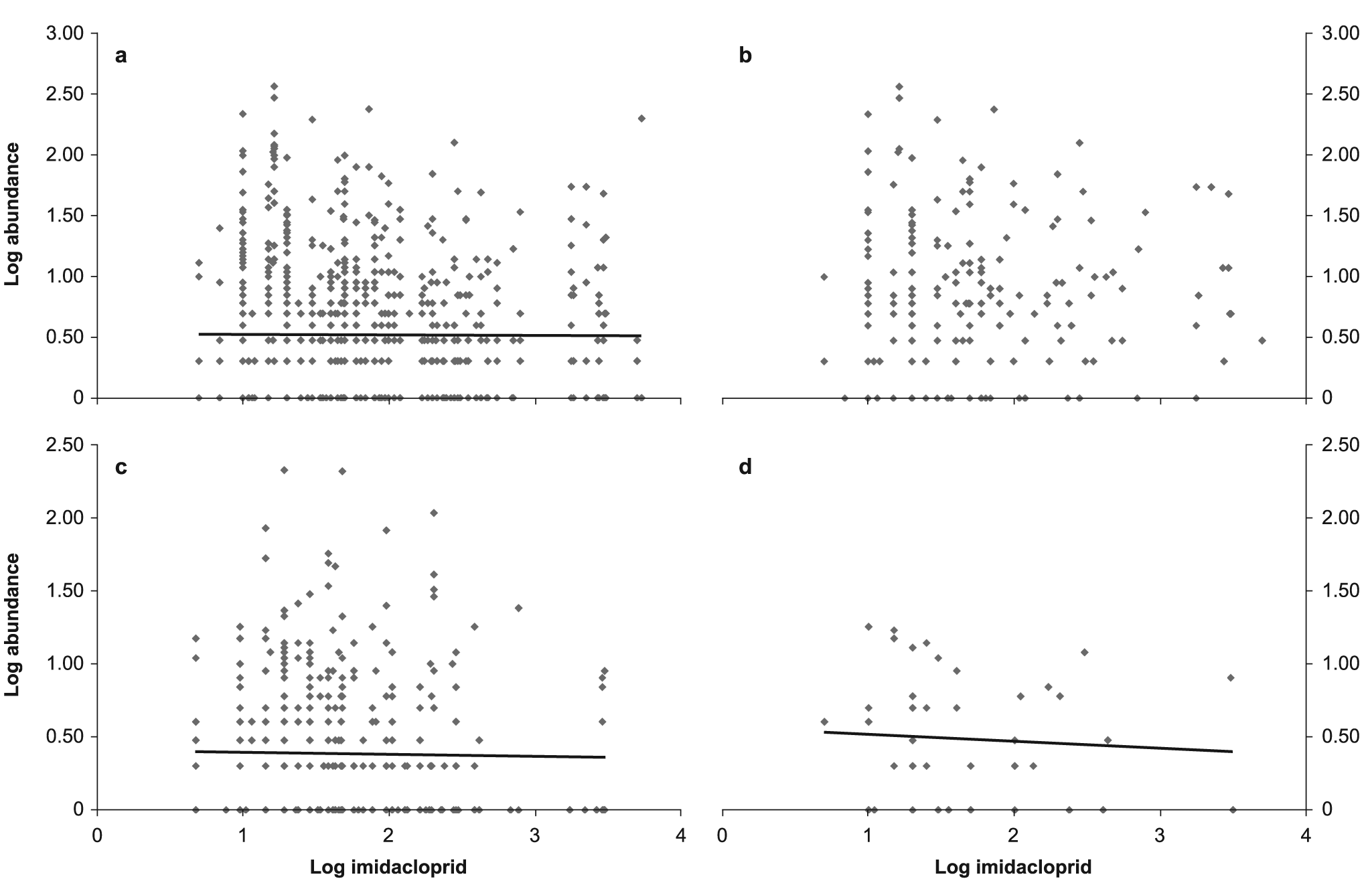

Supplement: Figure S5 — Relationship between log10 imidacloprid concentration and log10 Rhynchobdellae and Trichoptera abundance in surface water. a) Rhynchobdellae (P = 0.937), b) its most abundant species Helobdella stagnalis (P = 0.440), c) Trichoptera (P = 0.692), d) its most abundant species Mystacides longicornis (P = 0.651). (TIF) [file pone.0062374.s005.tif]

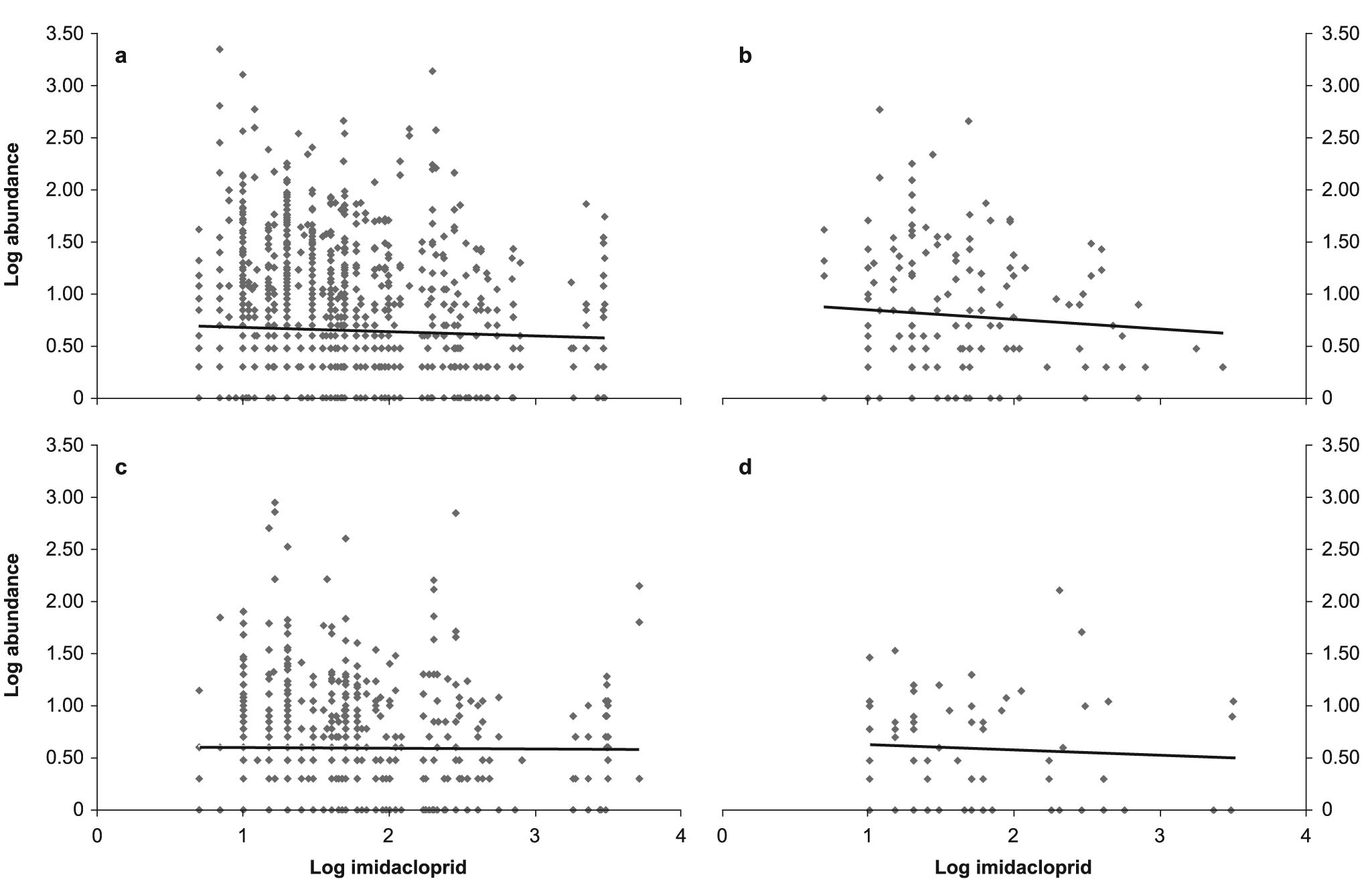

Supplement: Figure S6 — Relationship between log10 imidacloprid concentration and log10 Tubificidae and Veneroida abundance in surface water. a) Tubificidae (P = 0.210), b) its most abundant species Stylaria lacustris (P = 0.351), c) Veneroida (P = 0.776), d) its most abundant species Pisidium nitidum (P = 0.578). (TIF) [file pone.0062374.s006.tif]
