# Supplementary material for: Macro-Invertebrate Decline in Surface Water Polluted with Imidacloprid
Source: PLoS One. 2013 May 1;8(5):e62374. doi: 10.1371/journal.pone.0062374 (PMC3641074; doi:10.1371/journal.pone.0062374)
Supplement: Text S1 — The dataset and operations performed on the raw dataset of abundance data. (DOC) [file pone.0062374.s007.doc]

**Text S1 Macro-invertebrate decline in surface water polluted with imidacloprid**

***The dataset***

The combined dataset used for this study, is available for download from:

<http://www.nusap.org/PLoS_Van_Dijk_ea_2012_Data_supplement/Van_Dijk_ea_PLoS_data.zip>

This zip contains an Excel file, and a readme file to explain the layout of the Excel file.

***Operations performed on the raw dataset of abundance data***

The aquatic macro-invertebrate data for this study were obtained from 23 different Dutch water boards. However, the data files we received did not all have the same layout. Therefore the following operations were applied to standardize the data and make them suitable for our analysis.

- All the data were supplied in Microsoft Excel files. The files did not all have the same layout; in most files, the data were organized in columns, but in some a part of the data was organized in rows, while the columns and rows were not placed in the same order. The columns and rows contained location code, location coordinates, sampling date, species, abundance, order, as well as some other data. First, all the files that were partly organized in rows, were converted into files with only columns. Secondly, for each file, we selected the columns with the necessary data for our study, and then the columns were placed in the same order.
- In some files the location coordinates were missing; in that case, we requested a conversion file (with location codes and coordinates) from the water boards, and then converted the location codes into location coordinates and entered them in the respective columns of the data files.
- In some files the species names were missing and only species reference codes were given; in that case, we requested a conversion file (with species reference codes and species names) from the water boards, and then converted the species codes into species names in the respective data files.
- These conversions of data were executed with the Vertical Lookup function in Excel.
- Location coordinates were all converted into meters. Then the coordinates were checked to find out whether they were located within the Netherlands. There were a few impossible values for the coordinates, such as 100000 by 300000; the data associated with these locations were deleted. For one location the x- and y-coordinates had apparently been switched, because otherwise it would have been located outside of the Netherlands; this was corrected.
- Besides the species names, several files also contained the names of the orders the species belonged to, in a separate column. However, the grouping of the species into orders was not uniform, in some cases the given order was in fact not an order, and for many species there was no order name given. In some cases it was obvious that the species name was not spelled correctly, or spelled in different ways. With the use of the websites Taxonomicon (1) and Nederlands Soortenregister (2), the errors were corrected and missing order names were looked up. A conversion file was created, and with it, the species names were converted to order names which were entered in the respective column of the data files. Double records were filtered and deleted from the data files.
- Finally, the grouping into orders, and nomenclature, were checked once more (3); Tubificidae is not an order but a family, but is used here because the subclass Oligochaeta doesn’t have orders.

***References***

1. Brands SJ (comp.) 1989-present, *The Taxonomicon*, Universal Taxonomic Services, Zwaag, The Netherlands, <http://taxonomicon.taxonomy.nl/>, accessed late 2011 - early 2012.

2. Nederlands Soortenregister (Dutch Species Register) version 2.0, [www.nederlandsesoorten.nl](http://www.nederlandsesoorten.nl/), accessed late 2011 – early 2012

3. Beyens J, De Pauw N, Vannevel R (1991),

*Macro-invertebraten en waterkwaliteit: determineersleutels voor zoetwatermacro-invertebraten en methoden ter bepaling van de waterkwaliteit* (Stichting Leefmilieu, Antwerpen), 316 p.
